# Can Music Enhance Working Memory and Speech in Noise Perception in Cochlear Implant Users? Design Protocol for a Randomized Controlled Behavioral and Electrophysiological Study

**DOI:** 10.3390/audiolres14040052

**Published:** 2024-07-06

**Authors:** Kathrin Mertel, Andrew Dimitrijevic, Michael Thaut

**Affiliations:** 1 Music and Health Research Collaboratory (MaHRC), University of Toronto, Toronto, ON M5S 1C5, Canada; michael.thaut@utoronto.ca; 2 Sunnybrook Cochlear Implant Program, Sunnybrook Hospital, Toronto, ON M4N 3M5, Canada; andrew.dimitrijevic@utoronto.ca

**Keywords:** multimodal music training, cochlear implant, neurologic music therapy (NMT), speech understanding in noise, working memory, alpha oscillations, EEG

## Abstract

Background: A cochlear implant (CI) enables deaf people to understand speech but due to technical restrictions, users face great limitations in noisy conditions. Music training has been shown to augment shared auditory and cognitive neural networks for processing speech and music and to improve auditory–motor coupling, which benefits speech perception in noisy listening conditions. These are promising prerequisites for studying multi-modal neurologic music training (NMT) for speech-in-noise (SIN) perception in adult cochlear implant (CI) users. Furthermore, a better understanding of the neurophysiological correlates when performing working memory (WM) and SIN tasks after multi-modal music training with CI users may provide clinicians with a better understanding of optimal rehabilitation. Methods: Within 3 months, 81 post-lingual deafened adult CI recipients will undergo electrophysiological recordings and a four-week neurologic music therapy multi-modal training randomly assigned to one of three training focusses (pitch, rhythm, and timbre). Pre- and post-tests will analyze behavioral outcomes and apply a novel electrophysiological measurement approach that includes neural tracking to speech and alpha oscillation modulations to the sentence-final-word-identification-and-recall test (SWIR-EEG). Expected outcome: Short-term multi-modal music training will enhance WM and SIN performance in post-lingual deafened adult CI recipients and will be reflected in greater neural tracking and alpha oscillation modulations in prefrontal areas. Prospectively, outcomes could contribute to understanding the relationship between cognitive functioning and SIN besides the technical deficits of the CI. Targeted clinical application of music training for post-lingual deafened adult CI carriers to significantly improve SIN and positively impact the quality of life can be realized.

## 1. Introduction

People who suffer from hearing loss are restricted in their lifestyle, profession, and communication needs. If a hearing aid is no longer sufficient to understand speech, a cochlear implant (CI) can restore hearing and help with communication. This electronic hearing prosthesis has been used since the 1970s to significantly improve hearing, speech comprehension, and active participation in life for people of all ages who are severely hearing impaired or profoundly deaf. Although CI users can achieve high levels of speech understanding in quiet conditions, listening to speech in noisy (SIN) conditions, which is typical of everyday listening and music appreciation, still poses great challenges.

In summary, CI users commonly show a decreased perception of pitch, melody, harmony, and timbre, whereas rhythm perception is usually well preserved [1,2,3]. The limited delivery of spectral cues, poor frequency resolution in signal processing, electrode position in the cochlea, and individual duration of deafness may explain the low sound quality. CI manufacturers are aware of the issue of inadequate technical sound transmission and considerable effort is exerted for software optimization. Similarly, clinical researchers have been trying to reveal how specific musical training could support the development of new sound processing through a CI (e.g., [4,5,6]).

### 1.1. Music Training Improves Music Appreciation and Speech Understanding in CI Users

Listening to and creating music holds great cultural and social significance worldwide, bringing people together in communication and community. Despite the benefits of CIs, individuals with hearing impairments often refrain from participating in musical activities and their associated social environments.

For CI users, music processing may be significantly altered by auditory training that can promote brain plasticity in the auditory cortex despite the technological constraints of the CI [7]. Over the last 15 years, a variety of musical auditory training methods have been developed for CI users that have been shown to improve the recognition/processing of melodic lines, the identification of timbres, and the subjective perception of music (e.g., [8,9,10]). The degree of improvement has correlated with training intensity and regularity [11]. While these training programs range from six-week short-term auditory melodic training [8] to systematic active listening programs [12], and the use of online training resources [13], no systematic music rehabilitation strategy has been established. Additionally, Patient-Reported Outcomes (PROs), such as the Music-Related Quality of Life questionnaire [14], were utilized to evaluate individual music experiences across various real-life situations among different subgroups of CI patients [15,16,17]. While diverse music rehabilitation needs were identified, clear indications were not discernible to support the development of music rehabilitation programs for CI users [18,19,20]. Furthermore, training effects on music perception and appreciation, and effects on speech understanding in adult CI recipients should be reviewed with caution. Most of the present literature consists of correlational quasi-experimental studies with small sample sizes, considerable inter-individual variability, and moderate treatment effects, which prevents the formulation of definitive causal statements [21]. There is a lack of sufficient randomized controlled trials with large samples of hearing-impaired participants randomly assigned to an experimental and a control group to accurately assess the effects of music training.

Therefore, what remains to be done is increased research on the design and effect of music training programs on music appraisal and the effect of music training on speech understanding for CI users.

### 1.2. Music Training for Better Speech Understanding in Noise (SIN) with CI

Perceiving speech in noisy environments (e.g., phone conversations and social gatherings) is consistently compromised in individuals with hearing impairments, thus significantly reducing their quality of life. CI manufacturers have developed technological strategies like directional microphones, noise reduction algorithms, or synchronization with accessories to improve SIN comprehension.

Neuroscience research has shown various transfer effects of musical training on speech processing. Shared neural networks of music and language suggest that the plastic changes induced by musical training influence language processing and acquisition [22,23]. Both music and language must separate categorized sounds perceived within a continuous and complex stream. Over time and consistent exposure, the brain develops an internal temporal model to accurately anticipate forthcoming events [24]. Compared to speech, music places higher demands on pitch, temporal processing, and the analysis of auditory scenes. The latter is the leading process and refers to the capability to segregate between different but similar-sounding sources (e.g., one speaker within other speakers; the violin and the viola in a string quartet). When playing music, the brain performs at a high degree of precision in temporal and spectral synchronization and prediction [25]. Numerous studies starting from the 1990s have compared musicians and non-musicians and have shown significantly better auditory discrimination of basic linguistic structures (phoneme categorization, semantic discrimination, syntax, and prosody) in musicians. As an example, Schön et al. [26] showed that musical training improved melody processing not only in music perception but also in speech processing in normal-hearing participants.

After short-term musical training, Shahin [27] was able to demonstrate transfer effects for improved SIN in normal-hearing nonmusicians as well as enhanced speech understanding for hearing-impaired adults. Kang et al. [28] tested not only speech perception in quiet conditions but also included speech-in-noise tests after auditory musical training. They found that lower pitch discrimination thresholds, higher melody recognition scores, and timbre identification were correlated with SIN thresholds. Other groups documented enhanced SIN perception and auditory scene analysis ability in lifelong musicians [29,30]. Fowler et al. [31] verified the degree of music skills as a significant predictor of SIN in both the hearing and the CI population.

However, the mentioned and other related studies face multiple methodological limitations, such as small sample sizes, not being randomized or compared to an (age and condition-) matched control group, or not considering potential confounders and bias. Some have suggested that the benefits of musical training may be related to pre-existing factors like IQ and participants’ musical affinity rather than neuronal plasticity [32]. More data and valid research designs are required to further support the hypothesis that “better performance on complex auditory tasks such as music perception could generalize to better performance in other difficult auditory domains, such as speech in noise” [31].

In addition to training through analytical music listening, previous research has shown that playing/learning musical pieces integrates multiple sensory and motor systems. Playing an instrument creates an “action–perception link” between the motor and auditory systems of the brain [33,34]. While playing, auditory information must be constantly paired with motor activities (e.g., finger positions, lip tension) and sensory feedback. Continuous practice promotes auditory–motor plasticity and establishes strong connections between specific motor activities and precise corresponding acoustic information [35]. Furthermore, neuroscientific studies demonstrated an automatic coupling between auditory and motor systems when listening to rhythm and music (e.g., [36,37,38]). Enhanced activation in premotor areas was seen in both expert musicians and nonmusicians when they were listening to a melody they learned to play during short-term musical training [39]. Advanced rhythmic and musical skills seem to facilitate extracting temporal information of speech and thus improve understanding in the presence of background noise, as was shown in musicians [40].

Thus, auditory–motor (multi-modal) training may be more beneficial for CI users than purely auditory training [41]. Only sparse literature exists on the effects of multi-modal training on complex sound perception like vocal emotion recognition and pitch pattern recognition in CI rehabilitation. Chari et al. [42] tested the effect of a one-month computerized musical training on three groups of CI users (an auditory–motor group, an auditory-only group, and a no-training control group). Only the auditory–motor training group scored significantly better in the melodic contour identification task. These results support that short-term multi-modal music training significantly impacts pitch pattern recognition in CI users.

### 1.3. Cognitive Aspects of Speech Understanding

Besides fundamental auditory sensory processing skills, higher-level cognitive functions such as auditory working memory (WM) and selective attention are required to successfully understand spoken language and to communicate [43]. WM chunks extensive information into meaningful units, sets current information in context with previous information, and makes predictions. This cognitive process of remembering the beginning of a sentence to anticipate the end is fundamental to understanding language.

Characteristics of musical training (e.g., active music making, analytical music listening, composing) not only support neuroplastic changes in the auditory cortex but also shape executive functions [44,45,46] and successful learning [47]. Throughout the last decade, musicianship has been associated with improved auditory and enhanced higher-level cognitive functions like WM and selective attention [46]. WM plays a crucial role in both music perception and production when tracking chord progressions or memorizing scores to perform accurately and on time. As a result, musicians are superior at predicting acoustic events and understanding their statistical dependencies while listening or playing, thus demonstrating better verbal [48,49] and non-verbal WM [50,51].

The listed higher-level cognitive functions are required to successfully understand spoken language. WM, as the cortical temporal storage and processing system, even significantly predicts global speech comprehension (Pearson’s ρ = 0.30 to 0.52) [52]. Rönnberg and colleagues developed the Ease of Language Understanding framework (ELU) to describe the interplay between speech recognition and working memory [53,54,55]. In easy listening conditions, the input signal is matched immediately and effortlessly with the phonological representation of the auditory information in long-term memory. Understanding SIN distorts this matching process and requires higher-level cognitive functions for compensation: reliable bottom–up sensory encoding of target speech in the auditory system [56], compensatory sensorimotor integration [57], and top-–down functions like auditory WM and selective attention (e.g., [58,59,60,61]). This higher-level cognitive remedial processing is very effortful and lowers WM capacity.

The reduced spectrotemporal delivery of CIs may require additional cognitive resources for understanding speech in noisy environments. Consequently, CI users often exert more mental effort when listening to speech in noise, even when speech intelligibility is adjusted for equal performance compared to individuals with normal hearing [62,63]. For example, this increased listening effort has been linked to self-reported experiences of CI users during speech-in-noise tasks in Dimitrijevic et al. [64]. This heightened effort is associated with increased frustration, fatigue, and reduced concentration among CI users, impacting their performance at work or school and potentially leading to chronic stress and its negative effects on the mental quality of life (e.g., [65,66,67,68]).

Gray et al. [69] concluded in a mini-review that the characteristics of musical training as an integration of multiple sensory modalities and higher-order cognitive functions benefitted both WM performance and SIN perception in older adults. Just recently, Giallini et al. [70] confirmed a significant link between attention, cognition, and WM capacity in CI users. WM compensates for the degraded auditory signals provided by amplification systems and/or CI in noisy listening conditions but requires high cognitive resources [55,71]. Consequently, better speech performance was only reported in CI users with higher attentional resources [71]. Only a limited number of studies tested the effect of musical training on WM capacity with adult CI users. Most studies were conducted with CI children and showed particular benefits for WM. Long-term musical training improved auditory WM [72,73] or was superior to visual training for memory recall [74].

Considering the frequently described cognitive and auditory benefits due to the overlap in neural networks for processing speech and music, further evaluation of music-based training on WM performance in the speech perception of CI users is warranted.

### 1.4. Sentence Final Word Identification and Recall Test (SWIR)

As mentioned above, previous studies rarely included SIN tests to evaluate speech understanding in daily listening challenges and their neuronal components in hearing-impaired people. Ng and colleagues [75] developed the sentence final word identification and recall (SWIR) test, where participants are asked to immediately repeat sentences presented with background noise while simultaneously memorizing the last words for a memory recall after a unit of six sentences. The SWIR protocol enables the assessment of cognition when speech is simultaneously perceived and processed with hearing aids in background noise [75,76,77]. Importantly, SWIR sentences are understood by the participant since the signal-to-noise ratio (SNR) level is individually adjusted to 85% speech intelligibility, ensuring that recall performance reflects cognitive performance rather than audibility in the subsequent recall. Previous studies obtained average SNRs at +4.2 and +7.5 dB [77], which is representative of typical, real-life listening conditions. Additionally, the SWIR evaluates WM performance (memory recall of the sentence’s last works) like in realistic acoustic environments when the background noise and target speech come from multiple spatially separated locations. The dynamics of the mental rehearsal of six words in WM can be interpreted with the ELU framework. Lunner et al. [78] referred to the SWIR test as “suitable for testing hearing and hearing devices under more realistic and demanding everyday conditions than traditional speech-in-noise tests”. This study will apply a novel EEG approach to the SWIR test (SWIR-EEG).

### 1.5. Electrophysiological Measures Related to Speech Encoding, Working Memory, Attention, and Listening Effort

Most CI music perception studies emphasized CI behavioral performance on spectrotemporal acoustic features like pitch, timbre, melody perception, complex rhythm, and duration [1,79]. These spectrotemporal features are essential to parse speaker and background streams in SIN conditions. CIs distort auditory signals, which alter the perception in SIN conditions and may affect auditory WM abilities for their users. The analysis of electroencephalogram (EEG) patterns is one of the most common tools for electrophysiological investigations in CI users. It has millisecond time resolution and is safe to use in CI users as opposed to other neuroimaging modalities such as functional Magnetic Resonance Imaging. The CI-EEG literature has traditionally focused on passive listening paradigms designed for the pediatric population, primarily to objectively relate brain activity to their behavioral performance due to unreliable behavioral feedback. EEG measures during active listening requiring attention and working memory induce drastic neural oscillations not seen during passive listening [80]. EEG has a long history of quantifying cognitive-related brain potentials including neural oscillatory rhythms classically defined as canonical bands: delta (1–2 Hz), theta (3–6 Hz), alpha (8–12 Hz), beta (15–30 Hz), and gamma (35–40 Hz) [81]. It is well suited to study higher-level cognitive processing such as attention and working memory during SIN tasks as well as low-level sensory encoding. Even though attentive listening is associated with changes in nearly all canonical brain rhythms [80], the focus in this proposal will be on alpha and theta oscillatory activity, since these are the most commonly reported brain rhythms in the speech perception, attention, WM, and listening effort literature. Increased alpha activity was seen in the prefrontal cortex (PFC) during various WM situations and when listening in noisy conditions (e.g., [82,83,84,85]). Alpha activity in PFC is generally associated with WM updating during sensory processing [86,87] and simultaneously inhibiting non-involved brain regions [88]. Gray et al. [69] reported a positively corresponding decline in alpha-theta activity with distorted WM abilities in older persons. WM-related modulations of alpha-theta activity could illuminate problems in SIN perception [89,90]. Similarly, lower resting-state alpha and lower alpha-theta power were associated with high WM-load tasks [91], and an alpha-power reduction was seen in conditions with heightened sensitivity to distractions [92]. Additional support for this phenomenon has been shown in studies where hearing difficulty and attention increased with the level of background noise or vocoded speech [80,93,94]. Alpha oscillations seem to be particularly sensitive in WM paradigms when tested in hearing loss conditions. Petersen et al. [95] have observed an elevated alpha performance with increasing memory load in individuals with mild hearing loss but a significant drop in severe hearing loss conditions. The researchers assumed that a “cognitive limit” was reached when hearing under difficult conditions. This finding is consistent with other reports indicating that when cognitive resources for auditory perception are strained, less is available for cognitive processing [96,97,98]. At present, the neural correlates of profound hearing loss treated with a CI on WM and SIN conditions and how they intersect are still unclear. In addition, there is no documentation of possible changes in alpha-theta oscillatory activity associated with WM capacity and SIN perception after multi-modal musical training. Gray et al. [69] carried out a mini-review to investigate the relationship between musical training, WM, and SIN perception in healthy seniors (>65 years). They concluded that musical training may “preserve” WM-related alpha-theta activity and may benefit speech understanding in noisy conditions in older age. Dimitrijevic et al. [80] observed that alpha event-related synchronization and desynchronization occur as two separate components during SIN in normal hearing listeners. They also observed alpha oscillations in CI users during SIN screening tests and found that they are related to listening effort [64,99]. In addition to the cognitive evoked oscillatory changes described above, there is a large body of literature describing the sensory encoding of low-level sound features such as sound tone/speech onsets (REFS) [100], change responses [100], and more recently speech-neural tracking or coherence measures including the Temporal Response Function (TRFs) [101,102]. The TRF is a class of neural entrainment methods used to relate speech sounds to brain activity (reviewed in [103,104]). It represents a spatial filter that models brain responses for given acoustic features. The TRF shows enhancement during attention [105] and has been successfully used in CI users to quantify active listening [106,107]. This project will focus on the TRFs because the TRF response will be quantified to the speech envelopes using the SWIR sentence stimuli [80]. Detecting neural correlates of sensory and cognitive processing in CI users performing WM and SIN tasks after musical training may provide further insight into CI hearing processes than merely standard behavioral tests and may lead to more targeted clinical interventions in rehabilitation.

### 1.6. The Rationale for the Study

Given the cognitive and auditory perception benefits due to the overlap in neural networks for processing speech and music, further evaluation of music-based training on SIN in CI users is warranted.

As there is broad consensus that for both the normal hearing and hearing-impaired population, verbal WM functions are crucial in SIN, only a limited number of studies have described the effects of cognition and attention on speech perception in CI users.

In addition, improved auditory–motor coupling after music training seems to play a critical role in speech perception, especially in noisy conditions. This relationship has not been thoroughly investigated in people with CIs.

## 2. Research Questions

This study aims to investigate how speech in noise and working memory performance can be altered through focused multi-modal music training in post-lingual deafened adult CI users and gain a neurophysiological understanding of sensory and cognitive processes using EEG measures.

**Experiment 1**
**Training effects of musical training on Speech Understanding in Noise**
Research Questions:

(1) Does music training with separate focuses on pitch, timbre, and rhythm result in improvements in behavioral speech in noise measures (greater percent correct in SWIR task)?
(2) Does pitch- and timbre-based training result in greater improvements in behavioral speech in noise measures (greater percent correct in SWIR task) when compared to rhythm-based training?

**Experiment 2**
**Training effects of musical training on Working Memory Performance.**
Research Questions:

(1) Does music training with a focus on pitch, timbre, and rhythm result in improvements in behavioral working memory performance in noise measures (greater percent of recall in SWIR working memory task)?
(2) Does pitch and timbre-based training result in greater improvements in behavioral working memory performance in noise measures (greater percent of recall in SWIR working memory task) when compared to rhythm-based training?

**Experiment 3**
**EEG measures of musical training on Speech Understanding in Noise and Working Memory Performance.**
Research Question:

(1) Does music training alter EEG measures (alpha oscillation modulation) during the memory recall task of the SWIR?
(2) Does music training alter speech neural tracking during the sentence understanding task of the SWIR?

## 3. Materials and Methods

This project is a collaboration of the Music and Health Research Collaboration (MaHRC) of the University of Toronto and the CI Program of Sunnybrook Health Center in Toronto, ON, Canada.

### 3.1. Participants and Sample Size

Participants will be recruited from Canada’s largest cochlear implant program at Sunnybrook Health Science Center in Toronto. Included will be postlingually deafened persons aged from 18 to 80 years, both uni- and bilateral implanted with at least 1 year of CI experience, and having native or bilingual fluency in English. Exclusion criteria will be single-sided deafness (SSD), severe cognitive deficits, and neurologic (e.g., stroke) or psychiatric disease. Participants will be asked to sign consent forms for participation, approved by the Research Ethics Board at Sunnybrook and the University of Toronto at their first meeting, and will be compensated for each session throughout the duration of the study.

Using the G-Power program [108], a sample size of 18 people for each of the three experimental groups was calculated (0.7 based on the upper and lower ranges of alpha event-related desynchronization in the digits in noise to obtain at least a 10% change from the baseline). We aim for 27 participants per group to account for subject attrition.

### 3.2. EEG Recording and Study Procedure

This project will apply a novel EEG approach that provides behavioral and electrophysiological measures of the SWIR test (SWIR-EEG). All EEG data will be recorded using a 64-channel actiCHamp Brain Products recording system (Brain Products GmbH, Inc., Munich, Germany). The EEG will be segmented by sentence length. The sentence envelope will be used as a reference calculation of the temporal response function (TRF) using the mTRF toolbox [101]. Dynamic imaging of coherent sources (DICS) [109] will be used to determine speech–brain coherence, and the source of alpha power (8–12 Hz) will be determined [64].

For EEG analysis, CI-related artifacts will be reduced using independent component analysis (ICA). The team of the Sunnybrook CI brain lab developed a technique to identify and remove CI artifacts using cross-correlation (or TRFs) with the stimulus envelope and the ICA activations [110]. Time–frequency analysis for preprocessed continuous data will be performed using either BESA (Brain Electrical Source Analysis) [111] or FieldTrip [112] in addition to applying custom scripts in Matlab and FieldTrip for more advanced analyses (e.g., TRFs with speech envelopes, across trial beamformer correlations).

The overall study procedure will comprise two pre-train sessions (4 h each) at Sunnybrook CI brain lab, eight music training sessions (50 min each) at MaHRC, and one post-train session (4 h) over a period of 3 months per participant. Prior to the first pre-train session, participants will complete online quality-of-life questionnaires (Speech Spatial Qualities (SSQ) [113], Cochlear implant quality of Life-10 (CI-QoL-10), Cochlear Implant Quality of Life-35 (CI-QoL-35) [114]). Behavioral measurements of clinical speech perception will be assessed through the Sentence Matrix speech test [115]. In addition, the AzBio Sentence Test [116] will be applied in a 3D listening setup in the first EEG session and be followed by the SWIR-EEG procedure (see Section 3.3). The SWIR-EEG procedure will be repeated in the second pre-train session 4 weeks later. Two pre-train sessions are required to account for the learning effects and stability of the EEG measures and to determine behavioral and EEG imaging differences in the post-session. After the third and post-EEG session, the participants will fill out a short online “musical background and CI-music training feedback questionnaire” designed by the first author (KM) (see Supplementary Materials).

The participants will be randomly assigned into three separate training groups by single-blinded permuted-block randomization ABC [117]. Groups A (pitch) and B (timbre) will be the experimental groups because these tasks involve mostly pitch cues and CI users typically have problems with this type of task. Group C (rhythm) will serve as a control, given that CI users typically perform well on rhythm tasks, and therefore the effects of training are expected to be minimal [2].

The music training (4 weeks in total) will start in the week after the second pre-train session. The participants will take part in eight one-to-one practice sessions led by certified Neurologic Music Therapists, scheduled twice per week with each session lasting 50 min. All three groups will follow a gradually challenging music training paradigm for each of the three conditions. The exercise protocol was developed by the first author based on principles of the effect of multi-modal musical training on auditory and speech processing and will involve both active instrument playing and focused listening to recorded music.

**Figure 1:**
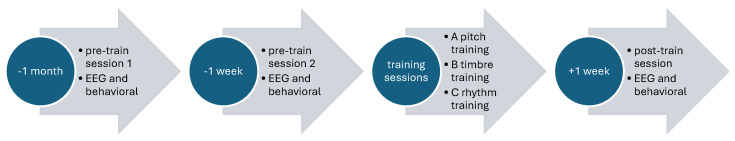
The overall outline of the experimental paradigm.

One post-train session will be scheduled one week after the music training to assess the short-term effects of the training by applying the online Sentence Matrix speech test [115] and the SWIR-EEG (see Figure 1 for the complete outline of the experimental paradigm).

### 3.3. The Procedure of the Sentence Final Word Identification and Recall Test (SWIR)

At first, the speech recognition threshold (SRT) for 85% correct sentence identification using HINT sentences will be determined by adaptively varying the signal-to-noise ratio of the sentence using speech-shaped noise. Each subject-specific SNR will be used in all of their repeated testing before and after music training. Participants will wear a 64-channel EEG cap and will be seated in a circular speaker ring array with a computer screen indicating instructions. The sentences will be presented by a speaker directly in front of the listener, while the other seven speakers will play speech babble. The test procedure will comprise twenty blocks of five HINT sentences in noise (SNR for 85% correct), where each block will include five trials of a list of six sentences. The participant will repeat the final word in each sentence (identification task). After each list, the participant will be asked to verbally repeat the last six words in any order (free recall task). The approximate time for this procedure will last about 50 min, including 10 min for the free recall task.

As mentioned before in Section 1.4 the SWIR test protocol enables the assessment of cognitive recall performance (WM) as indexed by the recall of the six final words of the sentence as well as subsequent speech understanding in noise.

### 3.4. Statistical Analysis

All data analysis including descriptive statistics will be performed using R [118]. Demographic data for each participant will include age, gender, etiology, handedness, years of education, and musical background. The grouping variable of interest will be “types of training” and will be coded into three categories: “A_pitch”, “B_timbre”, and “C_rhythm”. To test for differences after the training period within the three groups for each condition, one-way repeated measures ANOVA will be conducted. The 0.05 level of probability will be set as the level of statistical significance. The necessary assumptions for conducting a one-way repeated ANOVA should be met with the collected data. The participants in the three groups will be independent from each other, as they will be randomly assigned to the groups. The dependent variable “percentage of correctly identified words/sentence” is a ratio-measurement scale and will be tested to investigate if it is normally distributed before conducting the analysis. Levene’s test will be applied to test for homogeneity of variance among the groups. Should the one-way ANOVA analysis reveal that at least one group mean turns out as significantly different from the others; a post hoc test will be performed to detect which ones are significantly different from each other [119]. Differences between the groups will be analyzed with repeated measures ANOVA with three groups, an effect size of 80% power, and four repeated measurements. The 0.05 level of probability will be set as indicating statistical significance. Testing assumptions will be completed in the same format as the previous tests.

### 3.5. Instruments and Music Material

For pitch training, melody instruments like tone bars (range of two octaves), the glockenspiel (range of one octave), the metallophone (range of one octave), the piano, and the harp will be used.

For rhythm training, percussion instruments like hand drums, congas, triangles, double-row tambourines, and stand drums will be used. In addition, the piano will be used for presenting rhythm sequences.

For the timbre training, a variety of string, melodic, wind, percussion, and other instruments and different kinds of mallets will be employed. An established playlist of recorded music (source: youtube.com) of each instrument’s timbre, solo or in an ensemble, will be presented using a laptop and a Bose loudspeaker system. An additional playlist of wind instruments including flute, trumpet, trombone, saxophone, and clarinet as well as a training song composed by the principal author were recorded to be presented solo, in various combinations, and with band accompaniment.

## 4. Expected Outcome

If short-term focused multi-modal music training does enhance WM capacity and SIN in post-lingual deafened adult CI recipients, this study will provide behavioral and EEG neural correlates related to improvements after targeted music training. The results of this study could contribute to illuminating sensory–cognitive integration to support cognitive compensation during SIN perception besides the technological constraints of the CI device.

## 5. Conclusions

The impact of auditory–motor training on speech understanding in noise and its neural foundations in CI users has not been extensively researched. Meanwhile, studies on the effects of music training have primarily focused on individuals with normal hearing and extensive musical experience. Moreover, hearing rehabilitation following CI surgery varies globally, with some programs offering minimal training while others provide intensive auditory rehabilitation, with some including music training.

This project aims to assess both the neurophysiological and behavioral effects of multi-modal music training on SIN perception and WM in CI users. Expanding the understanding of sensory–cognitive music interventions and their direct neural effects on CI users is crucial for significant clinical implications.

The findings of this study could advance the clinical use of neurologic music training as an effective treatment for post-lingual deafened CI adults, enhancing their ability to understand speech in challenging noisy environments and thereby improving their quality of life.

## Supplementary Materials

The following supporting information can be downloaded at: https://www.mdpi.com/article/10.3390/audiolres14040052/s1, Survey “Questionnaire musical background and feedback of CI music training”.

## References

[1] Limb C.J., Roy A.T. (2014). Technological, biological, and acoustical constraints to music perception in cochlear implant users. Hear. Res..

[2] McDermott H.J. (2004). Music Perception with Cochlear Implants: A Review. Trends Amplif..

[3] Looi V., Gfeller K., Driscoll V. (2012). Music Appreciation and Training for Cochlear Implant Recipients: A Review. Semin. Hear..

[4] Magele A., Wirthner B., Schoerg P., Ploder M., Sprinzl G.M. (2022). Improved Music Perception after Music Therapy following Cochlear Implantation in the Elderly Population. J. Pers. Med..

[5] Driscoll V. (2012). The Effects of Training on Recognition of Musical Instruments by Adults with Cochlear Implants. Semin. Hear..

[6] Veltman J., Maas M.J.M., Beijk C., Groenhuis A.Y.M., Versnel H., Vissers C., Huinck W.J., Hoetink A.E. (2023). Development of the Musi-CI Training, A Musical Listening Training for Cochlear Implant Users: A Participatory Action Research Approach. Trends Hear..

[7] Fu Q.-J., Galvin J.J. (2008). Maximizing cochlear implant patients’ performance with advanced speech training procedures. Hear. Res..

[8] Lo C.Y., McMahon C.M., Looi V., Thompson W.F. (2015). Melodic Contour Training and Its Effect on Speech in Noise, Consonant Discrimination, and Prosody Perception for Cochlear Implant Recipients. Behav. Neurol..

[9] Smith L., Bartel L., Joglekar S., Chen J. (2017). Musical Rehabilitation in Adult Cochlear Implant Recipients with a Self-administered Software. Otol. Neurotol..

[10] Abdulbaki H., Mo J., Limb C.J., Jiam N.T. (2023). The Impact of Musical Rehabilitation on Complex Sound Perception in Cochlear Implant Users: A Systematic Review. Otol. Neurotol..

[11] Chen J.K.-C., Chuang A.Y.C., McMahon C., Hsieh J.-C., Tung T.-H., Li L.P.-H. (2010). Music Training Improves Pitch Perception in Prelingually Deafened Children with Cochlear Implants. Pediatrics.

[12] Firestone G.M., McGuire K., Liang C., Zhang N., Blankenship C.M., Xiang J., Zhang F. (2020). A Preliminary Study of the Effects of Attentive Music Listening on Cochlear Implant Users’ Speech Perception, Quality of Life, and Behavioral and Objective Measures of Frequency Change Detection. Front. Hum. Neurosci..

[13] Boyer J., Stohl J. (2022). MELUDIA—Online music training for cochlear implant users. Cochlear Implant. Int..

[14] Dritsakis G., Van Besouw R.M., Kitterick P., Verschuur C.A. (2017). A Music-Related Quality of Life Measure to Guide Music Rehabilitation for Adult Cochlear Implant Users. Am. J. Audiol..

[15] Adel Y., Nagel S., Weissgerber T., Baumann U., Macherey O. (2019). Pitch Matching in Cochlear Implant Users with Single-Sided Deafness: Effects of Electrode Position and Acoustic Stimulus Type. Front. Neurosci..

[16] D’Onofrio K.L., Caldwell M., Limb C., Smith S., Kessler D.M., Gifford R.H. (2020). Musical Emotion Perception in Bimodal Patients: Relative Weighting of Musical Mode and Tempo Cues. Front. Neurosci..

[17] Calvino M., Zuazua A., Sanchez-Cuadrado I., Gavilán J., Mancheño M., Arroyo H., Lassaletta L. (2024). Meludia platform as a tool to evaluate music perception in pediatric and adult cochlear implant users. Eur. Arch. Otorhinolaryngol..

[18] Lehmann A., Limb C.J., Marozeau J. (2021). Editorial: Music and Cochlear Implants: Recent Developments and Continued Challenges. Front. Neurosci..

[19] Mo J., Jiam N.T., Deroche M.L.D., Jiradejvong P., Limb C.J. (2022). Effect of Frequency Response Manipulations on Musical Sound Quality for Cochlear Implant Users. Trends Hear..

[20] Tahmasebi S., Gajȩcki T., Nogueira W. (2020). Design and Evaluation of a Real-Time Audio Source Separation Algorithm to Remix Music for Cochlear Implant Users. Front. Neurosci..

[21] Gfeller K., Young N.M., Iler Kirk K. (2016). Music as Communication and Training for Children with Cochlear Implants. Pediatric Cochlear Implantation.

[22] Koelsch S., Gunter T.C., Cramon V.D.Y., Zysset S., Lohmann G., Friederici A.D. (2002). Bach Speaks: A Cortical “Language-Network” Serves the Processing of Music. NeuroImage.

[23] Zatorre R.J., Belin P., Penhune V.B. (2002). Structure and function of auditory cortex: Music and speech. Trends Cogn. Sci..

[24] Pesnot Lerousseau J., Hidalgo C., Schön D. (2020). Musical Training for Auditory Rehabilitation in Hearing Loss. J. Clin. Med..

[25] Patel A.D. (2011). Why would Musical Training Benefit the Neural Encoding of Speech? The OPERA Hypothesis. Front. Psychol..

[26] Schön D., Magne C., Besson M. (2004). The music of speech: Music training facilitates pitch processing in both music and language. Psychophysiology.

[27] Shahin A.J. (2011). Neurophysiological Influence of Musical Training on Speech Perception. Front. Psychol..

[28] Kang R., Nimmons G.L., Drennan W., Longnion J., Ruffin C., Nie K., Won J.H., Worman T., Yueh B., Rubinstein J. (2009). Development and Validation of the University of Washington Clinical Assessment of Music Perception Test. Ear Hear..

[29] Zendel B.R., Alain C. (2012). Musicians experience less age-related decline in central auditory processing. Psychol. Aging.

[30] Zendel B.R., Alain C. (2013). The Influence of Lifelong Musicianship on Neurophysiological Measures of Concurrent Sound Segregation. J. Cogn. Neurosci..

[31] Fowler S.L., Calhoun H., Warner-Czyz A.D. (2021). Music Perception and Speech-in-Noise Skills of Typical Hearing and Cochlear Implant Listeners. Am. J. Audiol..

[32] McKay C.M. (2021). No Evidence That Music Training Benefits Speech Perception in Hearing-Impaired Listeners: A Systematic Review. Trends Hear..

[33] Barrett K.C., Ashley R., Strait D.L., Kraus N. (2013). Art and science: How musical training shapes the brain. Front. Psychol..

[34] Novembre G., Keller P.E. (2014). A conceptual review on action-perception coupling in the musiciansâ€^TM^ brain: What is it good for?. Front. Hum. Neurosci..

[35] Olthof B.M.J., Rees A., Gartside S.E. (2019). Multiple Nonauditory Cortical Regions Innervate the Auditory Midbrain. J. Neurosci..

[36] Zatorre R.J., Chen J.L., Penhune V.B. (2007). When the brain plays music: Auditory–motor interactions in music perception and production. Nat. Rev. Neurosci..

[37] Fujioka T., Zendel B.R., Ross B. (2010). Endogenous Neuromagnetic Activity for Mental Hierarchy of Timing. J. Neurosci..

[38] Grahn J.A. (2012). Neural Mechanisms of Rhythm Perception: Current Findings and Future Perspectives. Top. Cogn. Sci..

[39] Zendel B.R. (2022). The importance of the motor system in the development of music-based forms of auditory rehabilitation. Ann. N. Y. Acad. Sci..

[40] Slater J., Kraus N. (2016). The role of rhythm in perceiving speech in noise: A comparison of percussionists, vocalists and non-musicians. Cogn. Process.

[41] Herholz S.C., Zatorre R.J. (2012). Musical Training as a Framework for Brain Plasticity: Behavior, Function, and Structure. Neuron.

[42] Chari D.A., Barrett K.C., Patel A.D., Colgrove T.R., Jiradejvong P., Jacobs L.Y., Limb C.J. (2020). Impact of Auditory-Motor Musical Training on Melodic Pattern Recognition in Cochlear Implant Users. Otol. Neurotol..

[43] Van Knijff E.C., Coene M., Govaerts P.J. (2018). Speech understanding in noise in elderly adults: The effect of inhibitory control and syntactic complexity. Int. J. Lang. Commun. Disord..

[44] Bugos J.A., Perlstein W.M., McCrae C.S., Brophy T.S., Bedenbaugh P.H. (2007). Individualized Piano Instruction enhances executive functioning and working memory in older adults. Aging Ment. Health.

[45] Degé F., Schwarzer G. (2011). The Effect of a Music Program on Phonological Awareness in Preschoolers. Front. Psychol..

[46] Moreno S., Bidelman G.M. (2014). Examining neural plasticity and cognitive benefit through the unique lens of musical training. Hear. Res..

[47] Anders Ericsson K. (2008). Deliberate Practice and Acquisition of Expert Performance: A General Overview. Acad. Emerg. Med..

[48] Hanna-Pladdy B., Gajewski B. (2012). Recent and Past Musical Activity Predicts Cognitive Aging Variability: Direct Comparison with General Lifestyle Activities. Front. Hum. Neurosci..

[49] Parbery-Clark A., Strait D.L., Anderson S., Hittner E., Kraus N. (2011). Musical Experience and the Aging Auditory System: Implications for Cognitive Abilities and Hearing Speech in Noise. PLoS ONE.

[50] Hanna-Pladdy B., MacKay A. (2011). The relation between instrumental musical activity and cognitive aging. Neuropsychology.

[51] Francois C., Schon D. (2011). Musical Expertise Boosts Implicit Learning of Both Musical and Linguistic Structures. Cereb. Cortex.

[52] Daneman M., Merikle P.M. (1996). Working memory and language comprehension: A meta-analysis. Psychon. Bull. Rev..

[53] Rönnberg J., Lunner T., Zekveld A., Sörqvist P., Danielsson H., Lyxell B., Dahlström Ö., Signoret C., Stenfelt S., Pichora-Fuller M.K. (2013). The Ease of Language Understanding (ELU) model: Theoretical, empirical, and clinical advances. Front. Syst. Neurosci..

[54] Rönnberg J., Holmer E., Rudner M. (2019). Cognitive hearing science and ease of language understanding. Int. J. Audiol..

[55] Rudner M., Rönnberg J. (2008). The role of the episodic buffer in working memory for language processing. Cogn. Process.

[56] Du Y., Zatorre R.J. (2017). Musical training sharpens and bonds ears and tongue to hear speech better. Proc. Natl. Acad. Sci. USA.

[57] Du Y., Buchsbaum B.R., Grady C.L., Alain C. (2014). Noise differentially impacts phoneme representations in the auditory and speech motor systems. Proc. Natl. Acad. Sci. USA..

[58] Kraus N., Strait D.L., Parbery-Clark A. (2012). Cognitive factors shape brain networks for auditory skills: Spotlight on auditory working memory. Ann. N. Y. Acad. Sci..

[59] Escobar J., Mussoi B.S., Silberer A.B. (2020). The Effect of Musical Training and Working Memory in Adverse Listening Situations. Ear Hear..

[60] Puschmann S., Baillet S., Zatorre R.J. (2019). Musicians at the Cocktail Party: Neural Substrates of Musical Training during Selective Listening in Multispeaker Situations. Cereb. Cortex.

[61] Yeend I., Beach E.F., Sharma M. (2019). Working Memory and Extended High-Frequency Hearing in Adults: Diagnostic Predictors of Speech-in-Noise Perception. Ear Hear..

[62] Winn M.B. (2016). Rapid Release from Listening Effort Resulting from Semantic Context, and Effects of Spectral Degradation and Cochlear Implants. Trends Hear..

[63] Hughes K.C., Galvin K.L. (2013). Measuring listening effort expended by adolescents and young adults with unilateral or bilateral cochlear implants or normal hearing. Cochlear Implant. Int..

[64] Dimitrijevic A., Smith M.L., Kadis D.S., Moore D.R. (2019). Neural indices of listening effort in noisy environments. Sci. Rep..

[65] Shields C., Willis H., Nichani J., Sladen M., Kluk-de Kort K. (2022). Listening effort: *WHAT* is it, *HOW* is it measured and *WHY* is it important?. Cochlear Implant. Int..

[66] Sandi C., Haller J. (2015). Stress and the social brain: Behavioural effects and neurobiological mechanisms. Nat. Rev. Neurosci..

[67] Pichora-Fuller M.K. (2016). How Social Psychological Factors May Modulate Auditory and Cognitive Functioning during Listening. Ear Hear..

[68] Pichora-Fuller M.K., Kramer S.E., Eckert M.A., Edwards B., Hornsby B.W.Y., Humes L.E., Lemke U., Lunner T., Matthen M., Mackersie C.L. (2016). Hearing Impairment and Cognitive Energy: The Framework for Understanding Effortful Listening (FUEL). Ear Hear..

[69] Gray R., Sarampalis A., Başkent D., Harding E.E. (2022). Working-Memory, Alpha-Theta Oscillations and Musical Training in Older Age: Research Perspectives for Speech-on-speech Perception. Front. Aging Neurosci..

[70] Giallini I., Inguscio B.M.S., Nicastri M., Portanova G., Ciofalo A., Pace A., Greco A., D’Alessandro H.D., Mancini P. (2023). Neuropsychological Functions and Audiological Findings in Elderly Cochlear Implant Users: The Role of Attention in Postoperative Performance. Audiol. Res..

[71] Völter C., Oberländer K., Haubitz I., Carroll R., Dazert S., Thomas J.P. (2022). Poor Performer: A Distinct Entity in Cochlear Implant Users?. Audiol. Neurotol..

[72] Torppa R., Faulkner A., Huotilainen M., Järvikivi J., Lipsanen J., Laasonen M., Vainio M. (2014). The perception of prosody and associated auditory cues in early-implanted children: The role of auditory working memory and musical activities. Int. J. Audiol..

[73] Rochette F., Moussard A., Bigand E. (2014). Music Lessons Improve Auditory Perceptual and Cognitive Performance in Deaf Children. Front. Hum. Neurosci..

[74] Good A., Gordon K.A., Papsin B.C., Nespoli G., Hopyan T., Peretz I., Russo F.A. (2017). Benefits of Music Training for Perception of Emotional Speech Prosody in Deaf Children with Cochlear Implants. Ear Hear..

[75] Ng E.H.N., Rudner M., Lunner T., Pedersen M.S., Rönnberg J. (2013). Effects of noise and working memory capacity on memory processing of speech for hearing-aid users. Int. J. Audiol..

[76] Lunner T., Rudner M., Rosenbom T., Ågren J., Ng E.H.N. (2016). Using Speech Recall in Hearing Aid Fitting and Outcome Evaluation Under Ecological Test Conditions. Ear Hear..

[77] Ng E.H.N., Rudner M., Lunner T., Rönnberg J. (2015). Noise Reduction Improves Memory for Target Language Speech in Competing Native but Not Foreign Language Speech. Ear Hear..

[78] Lunner T., Alickovic E., Graversen C., Ng E.H.N., Wendt D., Keidser G. (2020). Three New Outcome Measures That Tap Into Cognitive Processes Required for Real-Life Communication. Ear Hear..

[79] Koelsch S., Wittfoth M., Wolf A., Müller J., Hahne A. (2004). Music perception in cochlear implant users: An event-related potential study. Clin. Neurophysiol..

[80] Dimitrijevic A., Smith M.L., Kadis D.S., Moore D.R. (2017). Cortical Alpha Oscillations Predict Speech Intelligibility. Front. Hum. Neurosci..

[81] Pfurtscheller G., Lopes Da Silva F.H. (1999). Event-related EEG/MEG synchronization and desynchronization: Basic principles. Clin. Neurophysiol..

[82] Bonnefond M., Jensen O. (2012). Alpha Oscillations Serve to Protect Working Memory Maintenance against Anticipated Distracters. Curr. Biol..

[83] Jensen O. (2002). Oscillations in the Alpha Band (9–12 Hz) Increase with Memory Load during Retention in a Short-term Memory Task. Cereb. Cortex.

[84] Jensen O., Mazaheri A. (2010). Shaping Functional Architecture by Oscillatory Alpha Activity: Gating by Inhibition. Front. Hum. Neurosci..

[85] Sauseng P., Klimesch W., Doppelmayr M., Pecherstorfer T., Freunberger R., Hanslmayr S. (2005). EEG alpha synchronization and functional coupling during top-down processing in a working memory task. Hum. Brain Mapp..

[86] Manza P., Hau C.L.V., Leung H.-C. (2014). Alpha Power Gates Relevant Information during Working Memory Updating. J. Neurosci..

[87] Misselhorn J., Friese U., Engel A.K. (2019). Frontal and parietal alpha oscillations reflect attentional modulation of cross-modal matching. Sci. Rep..

[88] Klimesch W., Sauseng P., Hanslmayr S. (2007). EEG alpha oscillations: The inhibition–timing hypothesis. Brain Res. Rev..

[89] StrauÃŸ A., WÃ¶stmann M., Obleser J. (2014). Cortical alpha oscillations as a tool for auditory selective inhibition. Front. Hum. Neurosci..

[90] Strauß A., Kotz S.A., Scharinger M., Obleser J. (2014). Alpha and theta brain oscillations index dissociable processes in spoken word recognition. NeuroImage.

[91] Rondina Ii R., Olsen R.K., Li L., Meltzer J.A., Ryan J.D. (2019). Age-related changes to oscillatory dynamics during maintenance and retrieval in a relational memory task. PLoS ONE.

[92] ElShafei H.A., Fornoni L., Masson R., Bertrand O., Bidet-Caulet A. (2020). Age-related modulations of alpha and gamma brain activities underlying anticipation and distraction. PLoS ONE.

[93] Wöstmann M., Herrmann B., Maess B., Obleser J. (2016). Spatiotemporal dynamics of auditory attention synchronize with speech. Proc. Natl. Acad. Sci. USA.

[94] Alain C., Du Y., Bernstein L.J., Barten T., Banai K. (2018). Listening under difficult conditions: An activation likelihood estimation meta-analysis. Hum. Brain Mapp..

[95] Petersen E.B., Wöstmann M., Obleser J., Stenfelt S., Lunner T. (2015). Hearing loss impacts neural alpha oscillations under adverse listening conditions. Front. Psychol..

[96] Arlinger S., Lunner T., Lyxell B., Kathleen Pichora-Fuller M. (2009). The emergence of Cognitive Hearing Science. Scand. J. Psychol..

[97] Lunner T., Rudner M., Rönnberg J. (2009). Cognition and hearing aids. Scand. J. Psychol..

[98] Pichora-Fuller M.K., Singh G. (2006). Effects of Age on Auditory and Cognitive Processing: Implications for Hearing Aid Fitting and Audiologic Rehabilitation. Trends Amplif..

[99] Paul B.T., Chen J., Le T., Lin V., Dimitrijevic A. (2021). Cortical alpha oscillations in cochlear implant users reflect subjective listening effort during speech-in-noise perception. PLoS ONE.

[100] Han J.-H., Dimitrijevic A. (2020). Acoustic Change Responses to Amplitude Modulation in Cochlear Implant Users: Relationships to Speech Perception. Front. Neurosci..

[101] Crosse M.J., Di Liberto G.M., Bednar A., Lalor E.C. (2016). The Multivariate Temporal Response Function (mTRF) Toolbox: A MATLAB Toolbox for Relating Neural Signals to Continuous Stimuli. Front. Hum. Neurosci..

[102] Crosse M.J., Zuk N.J., Di Liberto G.M., Nidiffer A.R., Molholm S., Lalor E.C. (2021). Linear Modeling of Neurophysiological Responses to Speech and Other Continuous Stimuli: Methodological Considerations for Applied Research. Front. Neurosci..

[103] Obleser J., Kayser C. (2019). Neural Entrainment and Attentional Selection in the Listening Brain. Trends Cogn. Sci..

[104] Verschueren E., Somers B., Francart T. (2019). Neural envelope tracking as a measure of speech understanding in cochlear implant users. Hear. Res..

[105] Fiedler L., Wöstmann M., Herbst S.K., Obleser J. (2019). Late cortical tracking of ignored speech facilitates neural selectivity in acoustically challenging conditions. NeuroImage.

[106] Xiu B., Paul B.T., Chen J.M., Le T.N., Lin V.Y., Dimitrijevic A. (2022). Neural responses to naturalistic audiovisual speech are related to listening demand in cochlear implant users. Front. Hum. Neurosci..

[107] Paul B.T., Uzelac M., Chan E., Dimitrijevic A. (2020). Poor early cortical differentiation of speech predicts perceptual difficulties of severely hearing-impaired listeners in multi-talker environments. Sci. Rep..

[108] Faul F., Erdfelder E., Lang A.-G., Buchner A. (2007). G*Power 3: A flexible statistical power analysis program for the social, behavioral, and biomedical sciences. Behav. Res. Methods.

[109] Hoechstetter K., Bornfleth H., Weckesser D., Ille N., Berg P., Scherg M. (2004). BESA source coherence: A new method to study cortical oscillatory coupling. Brain Topogr..

[110] Han J.-H., Zhang F., Kadis D.S., Houston L.M., Samy R.N., Smith M.L., Dimitrijevic A. (2016). Auditory cortical activity to different voice onset times in cochlear implant users. Clin. Neurophysiol..

[111] Research 7.1. http://www.besa.de/.

[112] Oostenveld R., Fries P., Maris E., Schoffelen J.-M. (2011). FieldTrip: Open Source Software for Advanced Analysis of MEG, EEG, and Invasive Electrophysiological Data. Comput. Intell. Neurosci..

[113] Gatehouse S., Noble W. (2004). The Speech, Spatial and Qualities of Hearing Scale (SSQ). Int. J. Audiol..

[114] McRackan T.R., Hand B.N., Velozo C.A., Dubno J.R., Cochlear Implant Quality of Life Development Consortium (2019). Cochlear Implant Quality of Life (CIQOL): Development of a Profile Instrument (CIQOL-35 Profile) and a Global Measure (CIQOL-10 Global). J. Speech Lang. Hear. Res..

[115] Hagerman B. (1982). Sentences for Testing Speech Intelligibility in Noise. Scand. Audiol..

[116] Spahr A.J., Dorman M.F., Litvak L.M., Van Wie S., Gifford R.H., Loizou P.C., Loiselle L.M., Oakes T., Cook S. (2012). Development and Validation of the AzBio Sentence Lists. Ear Hear..

[117] Efird J. (2010). Blocked Randomization with Randomly Selected Block Sizes. Int. J. Environ. Res. Public Health.

[118] R Core Team (2021). R: A Language and Environment for Statistical Computing.

[119] Plichta S.B., Kelvin E.A. (2011). Munro’s Statistical Methods for Health Care Research.

