# Supplementary material for: Can Music Enhance Working Memory and Speech in Noise Perception in Cochlear Implant Users? Design Protocol for a Randomized Controlled Behavioral and Electrophysiological Study
Source: Audiol Res. 2024 Jul 6;14(4):611–24. doi: 10.3390/audiolres14040052 (PMC11270222; doi:10.3390/audiolres14040052)
Supplement: Supplementary file 1 [file audiolres-14-00052-s001.zip › audiolres-3054819-supplementary.pdf]

Questionnaire musical background and feedback of CI music training

Dear participant,
Thank you for agreeing to participate in the CI music training study. After your music training, we would like you to ask a few questions. We are interested in your musical background and your experiences with the NMT music training at the Music and Health Collaboratory (MaHRC). Please select the answer that best applies to you. You can't go wrong! Your data will be treated confidentially and will only be used within this project.
If you have any questions, please reach out to us!

1. Musical background:

1.1 Please indicate your musical education or training and experience:

O Active professional musician before deafness
O Professionally trained, but played inconsistently before deafness
O No professional training, played frequently before deafness
O No professional training, played occasionally before deafness
O Moderate experience, no practical performance, basic musical knowledge available
O Little experience, no practical skills, little experience in listening
O No experience, no practical skills, no experience in listening

1.2 Did you have formal music training outside of school (instrumental or singing)?

O No
O Yes

1.3 How long did you have formal music training outside of school?

O less than 3 years
O more than 3 years

1.4 Did you sing before you got the CI?

O No
O Yes, for myself
O Yes, in a choir

1.5 Do you sing now?

O No
O Yes, on my own
O Yes, in a choir

1.6 Did you play or still play an instrument?

1.6.1 During childhood:

O No
O Yes
If yes, which instrument(s):____

1.6.2 Before hearing deficit:

O No
O Yes
If yes, which instrument(s):____

1.6.3 Before CI implantation:

O No
O Yes
If yes, which instrument(s):____

1.6.4 After CI-Implantation:

O No
O Yes
If yes, which instrument(s):____

2. Feedback on the CI music training program:

2.1 Which materials do/did you use for hearing training after your implantation?

Audio material like

O specific audio training material:____
O Audiobooks, podcasts
O music
O other:____

O I don’t/didn’t use specific audio material

Computer programs like

O exercise programs of Cochlea Implant manufacturers
O videos in the internet (e.g. YouTube)
O other:____

O I don’t/didn’t use computer resources

O no information

2.2 Which exercises during the CI music training have been helpful or did you like the most? Why?

____

2.3 Have there been exercises you did not like? For what reason?

O No

O Yes____

2.4 Did you miss something?

O No

O Yes____

2.5 How satisfied were you overall with the CI-Music training? Please circle the number of your selection:

Very unsatisfied neutral very satisfied

0 1 2 3 4 5 6

2.6 Were you able to benefit from the CI-Music training?

O No

O Yes, how? ____

- o got better
- o got worse

O other: ____

O no information

3. Personal rating of music listening and speech comprehension:

3.1 I rate my music-listening ability as

O very bad

O bad

O normal

O good

O very good

3.2 I enjoy listening to music

O very bad

O bad

O normal

O good

O very good

3.3 I understand speech

O very bad

O bad

O normal

O good

O very good

3.4 I rate my overall hearing ability as

O very bad

O bad

O normal

O good

O very good

Thank you very much!
